# LLCMDA: A Novel Method for Predicting miRNA Gene and Disease Relationship Based on Locality-Constrained Linear Coding

**DOI:** 10.3389/fgene.2018.00576

**Published:** 2018-11-28

**Authors:** Yu Qu, Huaxiang Zhang, Chen Lyu, Cheng Liang

**Affiliations:** School of Information Science and Engineering, Shandong Normal University, Jinan, China

**Keywords:** miRNA gene–disease relationship, similarity measure, association prediction, locality-constrained linear coding, label propagation

## Abstract

MiRNAs are small non-coding regulatory RNAs which are associated with multiple diseases. Increasing evidence has shown that miRNAs play important roles in various biological and physiological processes. Therefore, the identification of potential miRNA-disease associations could provide new clues to understanding the mechanism of pathogenesis. Although many traditional methods have been successfully applied to discover part of the associations, they are in general time-consuming and expensive. Consequently, computational-based methods are urgently needed to predict the potential miRNA-disease associations in a more efficient and resources-saving way. In this paper, we propose a novel method to predict miRNA-disease associations based on Locality-constrained Linear Coding (LLC). Specifically, we first reconstruct similarity networks for both miRNAs and diseases using LLC and then apply label propagation on the similarity networks to get relevant scores. To comprehensively verify the performance of the proposed method, we compare our method with several state-of-the-art methods under different evaluation metrics. Moreover, two types of case studies conducted on two common diseases further demonstrate the validity and utility of our method. Extensive experimental results indicate that our method can effectively predict potential associations between miRNAs and diseases.

## Introduction

MiRNAs are small non-coding regulatory RNAs. Since the first miRNA lin-4 (Lee et al., 1993) was found, a plenty of miRNAs have been discovered. Accumulating evidence has shown that miRNAs play a critical role in many biological processes, such as cell proliferation, differentiation, aging, and apoptosis (Ambros, 2004; Xu et al., 2004; Cheng et al., 2005; Miska, 2005; Huang et al., 2016). With the deepening of the research, researchers found that the dysfunctions of miRNAs are closely related to various diseases (Mei et al., 2016; Zou et al., 2016; Liao et al., 2018; Qu et al., 2018b; Tang et al., 2018), which sent an important signal to scientists from all around the world that exploring the associations between miRNAs and diseases is of great significance. Some experimental methods, such as PCR and Microarray (Thomson et al., 2007; Mohammadi-Yeganeh et al., 2013), have been able to successfully identify certain miRNAs related with diseases. However, it is unrealistic to use these traditional experimental methods to predict miRNA-disease associations at a large scale for their limitations of being time-consuming and expensive. To resolve this situation, multiple computational methods were proposed to efficiently uncover the potential associations between miRNAs and diseases.

Based on the assumption that miRNAs with similar functions are usually related to similar diseases (Zeng et al., 2016; Chen et al., 2017c), Jiang et al. (2010) proposed a network-based method to predict miRNA-disease associations using a hypergeometric distribution scoring system by constructing a miRNA functional similarity network and a human phenome-microRNAome network. Xuan et al. (2013) developed a method named HDMP based on weighted *k* most similar neighbors. They calculated miRNA functional similarity according to disease terms and disease phenotype similarity. In addition, the miRNAs within same families or clusters were assigned higher weights. Shi et al. (2013) performed random walk to predict miRNA-disease associations on protein–protein interaction (PPI) networks and achieved a satisfactory performance. Mørk et al. (2014) proposed a novel protein-driven method named miRPD to predict potential associations between miRNAs and diseases, where they presented a scoring scheme to efficiently predict and rank miRNA-disease associations. Considering that the global network-based methods could achieve better performance than local network-based methods, Chen et al. (2012) proposed a global similarity measure named RWRMDA. They applied random walk with restart to uncover miRNAs related with diseases on miRNA–miRNA functional similarity network. However, RWRMDA could not predict for diseases without any known related miRNAs. Li et al. (2017) proposed another method named MCMDA. In this method, they applied the matrix completion algorithm to update the known miRNA-disease associations matrix and predict the potential associations. Liu et al. (2017) also applied random walk to predict miRNA-disease associations on a heterogeneous network which was constructed by integrating multiple data sources. Similarly, Luo and Xiao (2017) used an imbalanced bi-random walk to predict miRNA-disease associations on a heterogeneous network consisting of miRNA functional similarity network, disease semantic network and known miRNA-disease association network. Chen et al. (2016a) presented another method WBSMDA to identify the associations between miRNAs and diseases by calculating Gaussian interaction profile kernel similarity for both miRNAs and diseases. Specifically, a within-score and a between-score were calculated and combined to gain a prediction score for each miRNA-disease pair. Using the same data, Chen et al. (2016b) presented HGIMDA which iteratively update an optimization function to uncover potential relations between miRNAs and diseases. Zeng et al. (2018) used structural consistency as an indicator to estimate the link predictability of the bilayer network and further predicted the potential associations between miRNAs and diseases based on Structural Perturbation Method (SPM). According to the lengths of different walks, Zou et al. (2015) introduced a path-based method using KATZ model and obtained reliable results. Similarly, You et al. (2017) proposed another effective path-based method named PBMDA. PBMDA also constructed a heterogeneous network and applied depth-first search algorithm to predict miRNA-disease associations. Although effective, the length of the paths in the searching process is limited to three. Qu et al. (2018a) presented a novel method SNMDA to identify potential diseases-related miRNAs based on sparse neighborhood and achieved comparable results. In recent years, several models based on machine learning have also been developed to predict the relationships between miRNAs and diseases (Chen et al., 2017b, 2018a,d). Based on semi-supervised learning framework, a model of Regularized Least Squares for MiRNA-Disease Association (RLSMDA) prediction was proposed by Chen and Yan (2014). Xiao et al. (2018) utilized graph-regularized non-negative matrix factorization to effectively predict for diseases without any related miRNAs based on heterogeneous omics data. Chen et al. (Zou et al., 2017) proposed an effective method ELLPMDA based on ensemble learning and link prediction. They integrated the results given by three classical similarity-based algorithms using ensemble learning. Li et al. (2018) presented a Kronecker kernel matrix dimension reduction (KMDR) model to predict miRNA-disease associations which integrates miRNA space and disease space into a larger miRNA-disease associations space. Chen et al. (2017a) proposed another model called MKRMDA that automatically optimizes the combination of multiple kernels. Recently, Chen et al. (2018b) presented EGBMMDA based on the model of extreme gradient boosting machine. Notably, EGBMMDA was the first decision tree learning-based model to uncover disease-related miRNAs and achieved favorable performance.

Although great efforts have been made to reliably predict miRNA-disease associations, there is still room for improvement. In this paper, we propose a novel method called LLCMDA for predicting miRNA-disease associations based on Locality-constrained Linear Coding (LLC). We apply four different cross-validation frameworks to comprehensively evaluate the performance of our method. The comparison results between LLCMDA and five state-of-the-art computational models demonstrate the utility of the proposed method. Besides, case studies on two common neoplasms further prove the effectiveness of our method. In summary, LLCMDA is an effective model for predicting potential miRNA–disease associations.

## Materials and methods

### Known miRNA-disease associations

HMDD (Li et al., 2014) is a database that records known experimentally-verified miRNA-disease associations, which contains 5,430 associations between 383 diseases and 495 miRNAs. For simplicity, an adjacency matrix *A* of dimension 495 ^*^ 383 is defined to describe the known miRNA-disease associations used in this paper. If miRNA *m*(*i*) has been confirmed to be related to *d*(*j*), *A* (*i, j*) = 1; otherwise *A* (*i, j*) = 0.

### MiRNA functional similarity

Wang et al. (2010b) proposed an informative measure to calculate miRNA functional similarities. Benefitting from previous researches, we downloaded miRNA similarity scores directly from http://www.cuilab.cn/files/images/cuilab/misim.zip. Similarly, we constructed a miRNA functional similarity matrix *FMS* to represent similarity scores, where *FMS* (*i, j*) represents the similarity score between miRNA *i* and miRNA *j*. A larger value indicates more similar function between two miRNAs.

### Disease semantic similarity

According to the Mesh descriptor, each disease can be described as a corresponding Directed Acyclic Network (DAG) (Wang et al., 2010a), i.e., DAG(*A*) = (*A, T*(*A*), *E*(*A*)), where *T*(*A*) is the node set including itself as well as its ancestor nodes, and *E*(*A*) represents the link set of *A*. Suppose disease *t* belongs to *T*(*A*), then the contribution of disease *t* to *A* can be calculated by:

(1) $$\begin{array}{l} \{\begin{array}{ll} D_{A}(t)=1 & if\;t=A\\ D_{A}(t)=\max\{0.5*D_{A}(t\prime )|t\prime \in child\;of\;t\} & if\;t\neq A \end{array} \end{array}$$

Besides, the semantic of *A* can be calculated by:

(2) $$\begin{array}{l} DV\left(A\right)=\sum_{t\in T_{\left(A\right)}}D_{A}\left(t\right) \end{array}$$

For disease *A* and *B*, the semantic similarity is calculated through the following formula:

(3) $$\begin{array}{l} S\left(A,B\right)=\frac{\sum_{t\in T\left(A\right)\cap T\left(B\right)}\left(D_{A}\left(t\right)+D_{B}\left(t\right)\right)}{DV\left(A\right)+DV\left(B\right)} \end{array}$$

where *t* is a common disease both in *T*(*A*) and *T*(*B*). *D*_*A*_(*T*)and *D*_*B*_(*T*)represent the contribution of disease *t* to the disease *A* and *B*, respectively. Therefore, for each disease pair, we can calculate their semantic similarity according to Equation (3). For convenience, we use an adjacency matrix *DSS* to denote the obtained semantic similarities for all disease pairs.

### Methods

**Figure 1:**
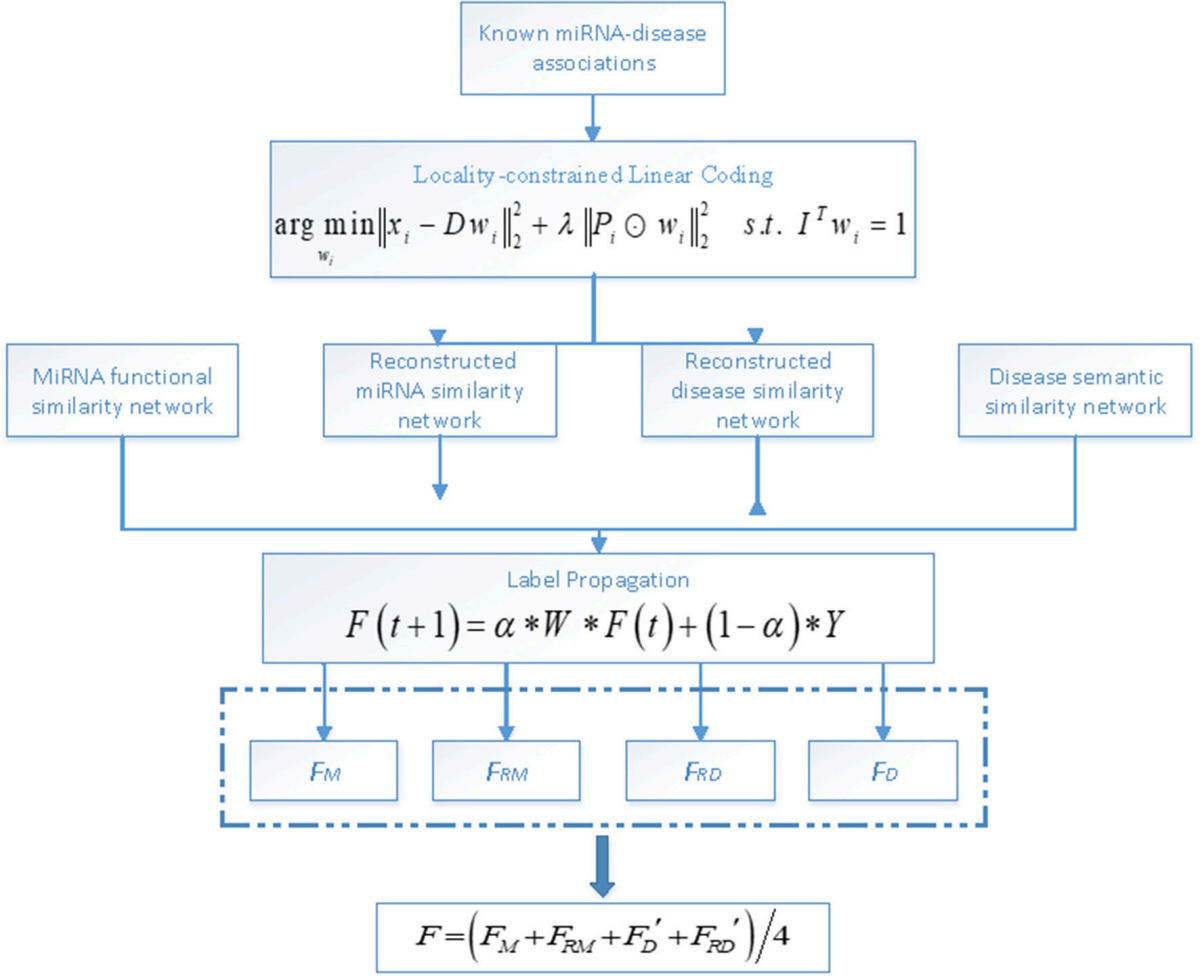
An overall workflow of LLCMDA to predict novel miRNA-disease associations.

In this paper, we predict potential associations between miRNAs and diseases based on LLC and label propagation. Specifically, the LLC algorithm is first used to reconstruct similarity networks for both miRNAs and diseases and then label propagation is applied on the similarity networks to obtain reliable predicted labels. An overall workflow of LLCMDA is illustrated in Figure 1.

#### Locality-constrained liner coding

Locality-constrained linear coding was first proposed by Wang et al. (2010b) and has been successfully applied to image classification. Compared with sparse representation, LLC is more computationally efficient and can preserve local information during the coding process (Saffari and Ebrahimi-Moghadam, 2015; Zhu et al., 2018). The objective function of LLC algorithm is defined as:

(4) $$\begin{array}{l} \underset{w_{i}}{\arg\min}||x_{i}-Dw_{i}|{|}_{2}^{2}+\lambda_{1}||P_{i}⊙w_{i}|{|}_{2}^{2}\;s.t.\;I^{T}w_{i}=1 \end{array}$$

Where *x*_*i*_ is the *i-*th sample, *D* represents a dictionary matrix and *P*_*i*_ is a local adapter vector representing the distances between the *i*-th sample and the other samples. λ_1_ is a regularization parameter. The sign of ⊙ denotes element-wise multiplication. Our goal is to find the optimized reconstructed similarities *w*_*i*_ for each sample *x*_*i*_. The Lagrangian function of Equation (4) can be obtained as follows:

(5) $$\begin{array}{l} \underset{w_{i}}{\arg\min}||x_{i}-Dw_{i}|{|}_{2}^{2}+\lambda_{1}||P_{i}⊙w_{i}|{|}_{2}^{2}+\lambda_{2}\left(I^{T}w_{i}-1\right) \end{array}$$

Where λ_2_ is the Lagrange multiplier. With simple algebra, the above equation can be further transformed into:

(6) $$\begin{array}{l} L\left(w_{i};\eta \right)=w_{i}^{T}Cw_{i}+\lambda_{1}w_{i}^{T}{\left\{diag\left(P_{i}\right)\right\}}^{2}w_{i}+\lambda_{2}\left(I^{T}w_{i}-1\right) \end{array}$$

where $C=\left(x_{i}I^{T}-D\right)\left(x_{i}I^{T}-D\right)$ and diag (*P*_*i*_) is a diagonal matrix whose (*j*,*j*)-th diagonal elements equals to the *j*-th element of vector *P*_*i*_. Specifically, we use the following formula to calculate the local distances between samples for *P*_*i*_:

(7) $$\begin{array}{l} P_{i}={\left\{P_{ij}\right\}}_{j=1,\ldots ,n}={\left\{\exp\left(\frac{{\left||x_{i}-x_{j}|\right|}_{2}}{\gamma }\right)\right\}}_{j=1,\ldots ,n} \end{array}$$

Where γ is a positive parameter controlling the bandwidth.

By taking the derivative of Equation (6) with respect to *w*_*i*_ and setting it to zero, we have:

(8) $$\begin{array}{l} \frac{\partial }{\partial w_{i}}L\left(w_{i};\eta \right)=0\Rightarrow Sw_{i}+\lambda_{2}1=0 \end{array}$$

where $S=2\left(C+\lambda_{1}{\left\{diag\left(P_{i}\right)\right\}}^{2}\right)$. By multiplying both sides of Equation (8) by 1^*T*^S^−1^ and considering the LLC constraint 1^*T*^*w*_*i*_ = 1, we can derive the optimal solution for *w*_*i*_ as follows:

(9) $$\begin{array}{l} \{\begin{array}{l} w_{i}=(C+{\left\{(diag(P_{i}))\right\}}^{2})\backslash I\\ w_{i}=w_{i}/I^{T}w_{i} \end{array} \end{array}$$

To obtain feature vectors as the input for LLC algorithm, we applied interaction profile to construct the feature vectors for miRNAs and diseases according to the known miRNA-disease associations (Zang and Zhang, 2012; Zhang et al., 2017). Specifically, the *i*-th row of adjacency matrix *A* represents the feature vector of miRNA *i* and the *j*-th column represents the feature vector of disease *j*. As a result, we can obtain two reconstructed similarity networks *RMS* and *RDS* for miRNAs and diseases according to Equation (9), respectively.

#### Label propagation

In this section, we adopt label propagation to obtain relevant scores of miRNA-disease pairs. In the process of label propagation, the known miRNA-disease associations are regarded as initial labels and label propagation is used to iteratively update labels (Zhang et al., 2018). Each point receives information not only from its neighbors but also its initial information. Here, we set a parameter α to control the rate. Therefore, the iteration equation on miRNA functional similarity network can be written as follows:

(10) $$\begin{array}{l} F_{M}\left(t+1\right)=\alpha *FMS*F_{M}\left(t\right)+\left(1-\alpha \right)*Y \end{array}$$

Here, *FMS* represents miRNA similarity network while *Y* represents the initial labels and *F*_*M*_ (0) = *Y*. We used Equation (10) to update the label information. When the iteration equation converges, *F*_*M*_(*t*+1) is regarded as the relevant score matrix. Therefore, we can sort the miRNAs by relevant scores for each disease. According to previous studies (Zhou et al., 2003), *FMS* is guaranteed to converge if it is properly normalized as follows:

(11) $$\begin{array}{l} FMS=D^{-1/2}*FMS*D^{1/2} \end{array}$$

where *D* is a diagonal matrix, the values on the diagonal correspond to the sum of all elements in each row. Similarly, we apply label propagation on the other three similarity networks *RMS, DSS*, and *RDS* to obtain three relevant score matrixes *F*_*RM*_, *F*_*D*_, and *F*_*RD*_. At last, we integrate the four prediction results and take the average as the final output *F*.

(12) $$\begin{array}{l} F=\left(F_{M}+F_{RM}+F_{D}^{\prime }+F_{RD}^{\prime }\right)/4 \end{array}$$

#### Implementation details

LLCMDA is implemented in MATLAB under the MATLAB R2016b programming environment. All the experiments are performed on a desktop with an i7-6700 3.40 GHz CPU and 16G RAM. The source code of LLCMDA is freely available at: https://github.com/misitequ/LLCMDA.

## Results

### Evaluation

In this section, three cross-validation frameworks are applied to test the performance of our algorithm: global LOOCV, local LOOCV, and five-fold cross-validation. In the framework of global LOOCV, each known miRNA-disease association is left out in turn as a test sample, and the other associations are regarded as training samples. After prediction, each miRNA-disease pair would obtain a score accordingly. If its ranking is higher than a given threshold, the prediction is regarded as a successful prediction. In the framework of local LOOCV, a disease is given in advance and then each miRNA associated with this disease is left out in turn as a test sample while the rest of miRNAs associated with the disease are set as seed samples. The only difference between global LOOCV and local LOOCV is that whether we simultaneously consider the candidates from all diseases (Chen et al., 2018a,c). Five-fold cross validation is also implemented to verify the utility of our method. Concretely, the 5,430 known associations are randomly divided into five subsets, each subset is taken as test samples in turn and the others are considered as training samples. To avoid the bias caused by random division of samples, we repeat five-fold cross-validation 20 times and take the average as the final result. Receiver-Operating Characteristics (ROC) curves are plotted by calculating True Positive Rate (TPR) and False Positive Rate (FPR) at varying thresholds. We then calculate the Area Under the ROC Curve (AUC) to quantitatively evaluate the performance of prediction models. AUC = 1 means the model is perfect while AUC = 0.5 denotes a random prediction.

**Figure 2:**
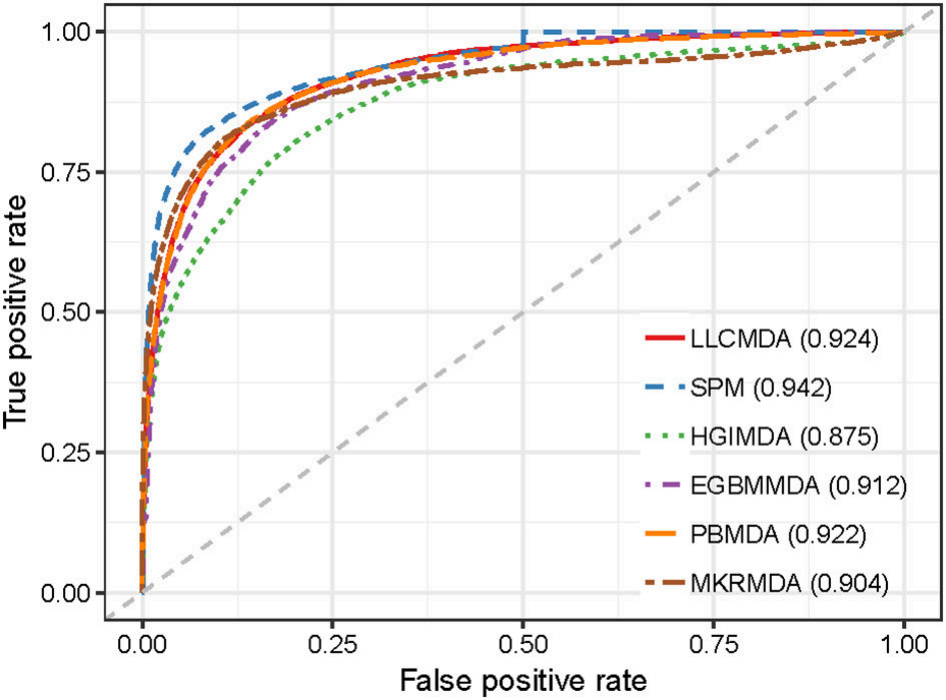
The comparison results between LLCMDA and other four methods (SPM, HGIMDA, EGBMMDA, PBMDA, MKRMDA) in terms of global LOOCV.

**Figure 3:**
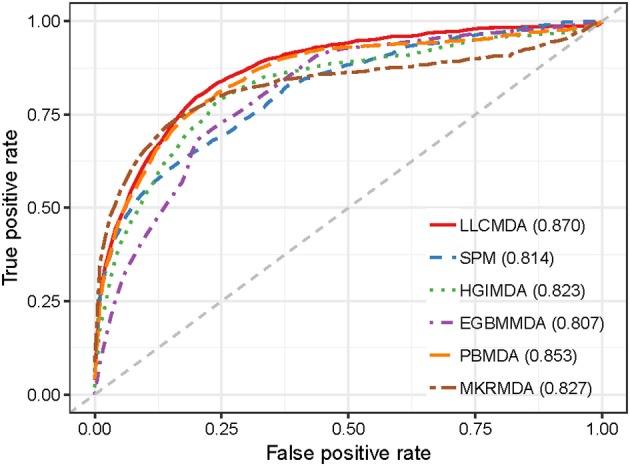
The comparison results between LLCMDA and other four methods (SPM, HGIMDA, EGBMMDA, PBMDA, MKRMDA) in terms of local LOOCV.

**Figure 4:**
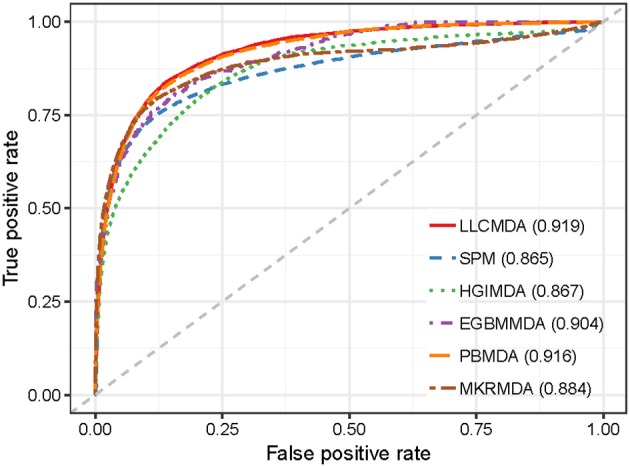
The comparison results between LLCMDA and other four methods (SPM, HGIMDA, EGBMMDA, PBMDA, MKRMDA) in terms of five-fold cross-validation.

As a result, LLCMDA obtained the AUCs of 0.924, 0.870, and 0.919 in global LOOCV, local LOOCV, and five-fold cross-validation, respectively. To further illustrate the effectiveness of our algorithm, we compared LLCMDA with five state-of-the-art methods, i.e., SPM, HGIMDA, PBMDA, MKRMDA, EGBMMDA. In the framework of global LOOCV, SPM, HGIMDA, PBMDA, MKRMDA, and EGBMMDA achieved AUCs of 0.942,0.875, 0.922, 0.904, and 0.912 (Figure 2). In local LOOCV, the AUCs obtained by SPM, HGIMDA, PBMDA, MKRMDA, and EGBMDA were 0.814, 0.823, 0.853, 0.827, and 0.807 (Figure 3). In addition, they obtained AUC-values of 0.865, 0.867, 0.916, 0.884, and 0.904 in five-fold cross-validation (Figure 4), respectively. As can be seen from the results, the AUCs of LLCMDA were higher than that of the other methods in all three cross-validation frameworks except the global LOOCV. In conclusion, our method is reliable to predict the potential miRNA-disease associations.

**Figure 5:**
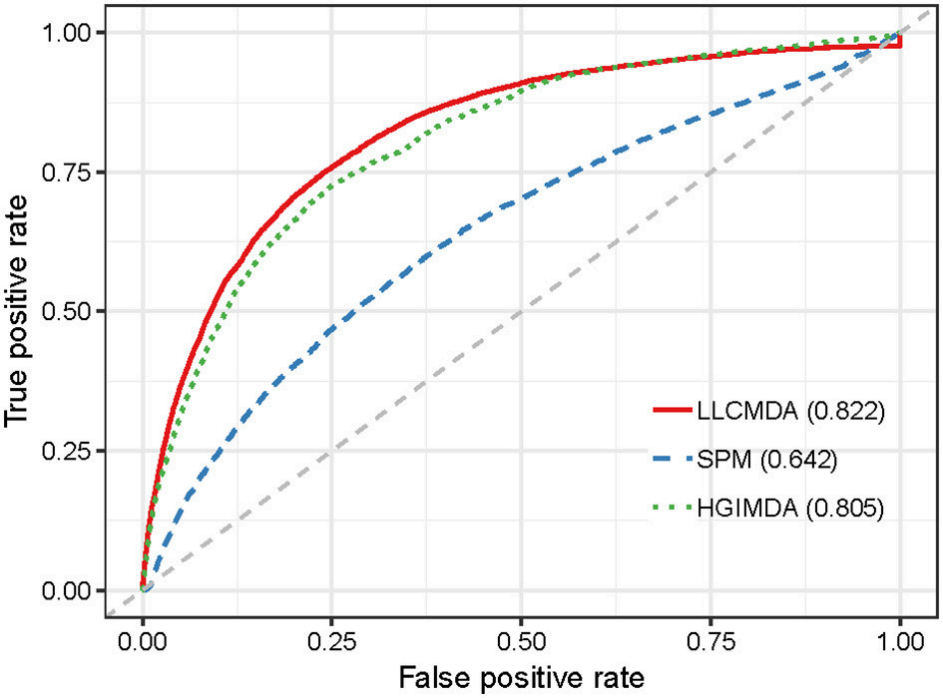
The comparison results between LLCMDA, SPM and HGIMDA in terms of LODOCV.

To further test the performance of our method in predicting new associations for diseases without any known related miRNAs, we adopted another evaluation metric called Leave One Disease Out Cross Validation (LODOCV) (Fu and Peng, 2017). In particular, we removed all the associated miRNAs for a given disease and then prioritized all the candidate miRNAs based on the known associations of other diseases. LODOCV is considerably more stringent than the afore mentioned cross-validation frameworks since there is no prior association information available for the given disease. We also compared LLCMDA with the five state-of-the-art methods in terms of the AUC-values. As shown in Figure 5, LLCMDA achieved the highest AUC-value of 0.822 in LODOCV framework. Here, we only demonstrate the performances of LLCMDA, SPM, and HGIMDA in the figure as the AUC-values obtained by the other three methods were lower than 0.6. The experimental results indicate that LLCMDA has better generalization ability in predicting new miRNA-disease associations.

### Parameter analysis

**Figure 6:**
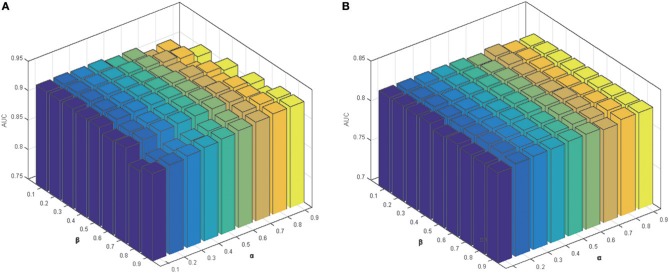
The parameter effects on the prediction performance in: **(A)** five-fold cross-validation; **(B)** LODOCV.

Parameter α was used to control the rate of the initial labels on the prediction results for miRNA in Equation (10). Similarly, we used another parameter β to control the effects of initial labels for diseases. To explore the impact of the two parameters, we set different values (0.1–0.9) for both parameters to obtain the prediction results in five-fold cross-validation and LODOCV frameworks (Figure 6). It can be seen that parameter α and β only have minor effects on the final prediction accuracies. Similar trends were also observed in global LOOCV and local LOOCV. Consequently, both parameters were set to 0.5.

### Case study

In recent years, substantial evidence suggests that miRNAs are associated with various neoplasms, such as breast neoplasms, lung neoplasms, and etc. Here, we conducted two types of case studies to validate the utility of LLCMDA on two common neoplasms, lung neoplasms and lymphomas. The case studies on other diseases can be found at https://github.com/misitequ/LLCMDA. We selected the top 50 miRNAs predicted by our model for each disease. The prediction results were then verified by another three databases, i.e., mir2disease (Jiang et al., 2009), dbDEMC (Yang et al., 2017), and miRwayDB (Das et al., 2018), which all record experimentally-validated miRNA-disease associations.

Lung neoplasms is one of the malignant tumors with the fastest increase in morbidity and mortality and the greatest threat to human health and life (Yanaihara et al., 2006). Therefore, there is an urgent need to identify prognostic and predictive markers for early detection. We used our method to uncover the potential miRNAs and listed the top 50 predicted candidate miRNAs. As a result (Table 1), 46 out of the top 50 miRNAs were verified to be associated with lung neoplasms by at least one database from Mir2disease, dbDEMC, and miRwayDB. For instance, studies have shown that hsa-mir-16(1st in Table 1) and hsa-mir-429 (3rd in Table 1) are closely related to the diagnosis and treatment of lung cancer (Reid et al., 2013; Ren et al., 2016).

**Table 1:** Top 50 predicted miRNAs associated with Lung Neoplasms based on known associations in HMDD.

**miRNA (1–25)**	**Evidence**	**miRNA (26–50)**	**Evidence**
hsa-mir-16	I;II;III;	hsa-mir-488	I;
hsa-mir-106b	I;	hsa-mir-376c	I;III;
hsa-mir-429	I;II;	hsa-mir-451	I;II;
hsa-mir-195	I;II;	hsa-mir-302d	I;
hsa-mir-141	I;II;III;	hsa-mir-449a	I;
hsa-mir-130a	I;II;	hsa-mir-520b	I;
hsa-mir-15a	I;II;III;	hsa-mir-139	I;II;
hsa-mir-151	unconfirmed;	hsa-mir-193b	I;
hsa-mir-302a	I;	hsa-mir-383	I;
hsa-mir-373	I;	hsa-mir-194	I;III;
hsa-mir-20b	I;	hsa-mir-149	I;
hsa-mir-296	unconfirmed;	hsa-mir-10a	I;III;
hsa-mir-302c	I;	hsa-mir-452	I;III;
hsa-mir-92b	I;	hsa-mir-491	I;
hsa-mir-339	I;II;	hsa-mir-144	I;III;
hsa-mir-372	I;II;	hsa-mir-520c	unconfirmed;
hsa-mir-28	I;	hsa-mir-449b	I;
hsa-mir-23b	I;	hsa-mir-484	I;
hsa-mir-367	I;	hsa-mir-299	unconfirmed;
hsa-mir-99b	I;	hsa-mir-204	I;II;
hsa-mir-130b	I;	hsa-mir-382	I;
hsa-mir-15b	I;II;	hsa-mir-129	I;
hsa-mir-99a	I;II;III;	hsa-mir-432	I;
hsa-mir-215	I;	hsa-mir-301b	I;
hsa-mir-342	I;	hsa-mir-423	II;

*I, II and, III represent dbDEMC, miR2Disease, and miRwayDB, respectively. The first and third columns record the 1–25 and 26–50 related miRNAs, respectively*.

To verify the potency of our method on real datasets, we conducted the second type of case study where we used older version of HMDD (v 1.0) as input to predict potential associations and test whether LLCMDA could uncover the newly-added ones in the latest version of HMDD (v 2.0). Specifically, HMDD v 1.0 contains 1,395 associations between 271 miRNAs and 137 diseases (Zhao et al., 2018). Here, we chose Lymphomas for validation. As shown in Table 2, 48 out of the top 50 candidate miRNAs have been confirmed by dbDEMC, miR2Disease or/and miRwayDB. In particular, 31 miRNAs were found in HMDD 2.0. Taken together, these evidence further showed that our prediction method can effectively predict potential associations between miRNAs and diseases.

**Table 2:** Top 50 predicted miRNAs associated with Lymphomas based on known associations in the older version of HMDD.

**miRNA (1–25)**	**Evidence**	**miRNA (26–50)**	**Evidence**
hsa-mir-21	HMDDv2.0;I;II;III;	hsa-mir-668	HMDD;I;
hsa-mir-155	HMDDv2.0;I;II;III;	hsa-mir-339	I;
hsa-mir-221	HMDDv2.0;I;II;	hsa-mir-143	HMDDv2.0;I;
hsa-mir-146a	HMDDv2.0	hsa-mir-10a	HMDDv2.0
hsa-mir-222	HMDDv2.0;I;	hsa-mir-30d	I;II;
hsa-let-7e	HMDDv2.0;I;II;	hsa-mir-187	I;
hsa-let-7d	HMDDv2.0;I;	hsa-mir-205	I;
hsa-mir-34a	HMDDv2.0;I;	hsa-mir-93	HMDDv2.0;I;
hsa-let-7g	HMDDv2.0;I;	hsa-mir-34c	HMDDv2.0;I;
hsa-mir-200b	HMDDv2.0;I;	hsa-mir-15b	unconfirmed;
hsa-let-7b	HMDDv2.0;I;	hsa-mir-429	I;
hsa-mir-223	HMDDv2.0;I;	hsa-mir-142	unconfirmed;
hsa-mir-29a	HMDDv2.0;I;	hsa-mir-25	HMDDv2.0;III;
hsa-mir-29c	HMDDv2.0;I;	hsa-mir-106a	I;
hsa-mir-145	HMDDv2.0;I;II;III;	hsa-mir-373	I;II;
hsa-let-7c	HMDDv2.0;I;II;	hsa-mir-200c	HMDDv2.0;I;
hsa-let-7i	HMDDv2.0;I;	hsa-mir-302c	HMDDv2.0;I;III;
hsa-mir-146b	I;	hsa-mir-34b	I;
hsa-mir-127	HMDDv2.0;II;	hsa-mir-302d	I;II;
hsa-mir-106b	I;III;IV;	hsa-mir-191	I;
hsa-mir-200a	HMDDv2.0;I;II;	hsa-mir-150	I;
hsa-mir-126	HMDDv2.0;I;	hsa-mir-30e	HMDDv2.0;I;II;III;
hsa-mir-141	I;	hsa-mir-367	HMDDv2.0;I;
hsa-mir-135b	HMDDv2.0;I;III;	hsa-mir-215	I;
hsa-mir-125a	HMDDv2.0;I;II;III;	hsa-mir-19b	I;

*I, II, and III represent dbDEMC, miR2Disease, and miRwayDB, respectively. The first and third columns record the 1–25 and 26–50 related miRNAs, respectively*.

## Discussion

Nowadays, identifying potential disease-associated miRNAs could provide new insights into the role of miRNA as valuable biomarkers for clinical measure, diagnosis and treatment. However, it is impossible to predict the associations between miRNA-disease relying on traditional experimental-based methods. Consequently, great numbers of computational methods have been proposed to solve this challenging problem in recent years. In this paper, we presented a novel method to predict potential miRNA-disease associations based on locality-constrained liner coding. We first applied LLC algorithm to reconstruct similarity networks for miRNAs and diseases. The label propagation was then applied on the similarity networks to retrieve relevant scores for each miRNA-disease association. The final results were calculated as the average of the predicted results from both miRNA space and disease space, respectively. To comprehensively verify the performance of our method, we compared LLCMDA with five state-of-the-art computational model under four different cross-validation frameworks. The experimental results demonstrated powerful evidence that our method could effectively predict miRNA-disease associations. In addition, case studies on two common diseases also gave a strong confirmation to the prediction ability of our method.

The success of our method is mainly due to the following two reasons. First, the reconstructed similarity networks for both miRNAs and diseases are more robust as the LLC algorithm regards the local information in the coding process. Second, we applied label propagation on the reconstructed similarity networks as well as the original similarity networks to calculate reliable relevant scores for the final output. Nonetheless, more informative data sources should be integrated into our model to further improve the prediction performance. Besides, the final outcome was simply taken as the average from the prediction scores from different similarity networks, which may lead to sub-optimal results. Therefore, a more appropriate way to incorporate the prediction results needs to be put forward.

## Author contributions

YQ and CLi conceived the study and planned experiments. YQ and HZ designed the algorithm and implemented. CLy and HZ performed data analysis. YQ and CLi drafted the manuscript. All authors read and approved the final manuscript.

### Conflict of interest statement

The authors declare that the research was conducted in the absence of any commercial or financial relationships that could be construed as a potential conflict of interest.
